# In-depth metataxonomic investigation reveals low richness, high intervariability, and diverse phylotype candidates of archaea in the human urogenital tract

**DOI:** 10.1038/s41598-023-38710-9

**Published:** 2023-07-20

**Authors:** Yeon Bee Kim, Tae Woong Whon, Joon Yong Kim, Juseok Kim, Yujin Kim, Se Hee Lee, Seong-Eun Park, Eun-Ju Kim, Hong-Seok Son, Seong Woon Roh

**Affiliations:** 1 Kimchi Functionality Research Group, World Institute of Kimchi, Gwangju, 61755 Republic of Korea; 2 Interdisciplinary Program in Agricultural Genomics, College of Agriculture and Life Sciences, Seoul National University, Seoul, 08826 Republic of Korea; 3 Microbiome Research Team, LISCure Biosciences Inc, Gyeonggi-do, 13486 Republic of Korea; 4 Department of Biotechnology, College of Life Sciences and Biotechnology, Korea University, Seoul, 02841 Republic of Korea

**Keywords:** Microbiology, Archaea, Microbial communities

## Abstract

The urogenital microbiota is the potential principal factor in the pathophysiology of urinary tract infection and the protection of urinary tract health. Little is known about the urogenital archaeome although several reports have indicated that the archaeomes of various regions of the human body are associated with health. Accordingly, we aimed to determine the presence and diversity of archaeomes in the human urogenital tract. To explore the urogenital archaeome, voided urine specimens from 373 asymptomatic Korean individuals were used. No difference was observed in body mass index, age, or gender, according to presence of archaea. Analysis of archaeal 16S rRNA gene amplicons of archaea positive samples consisted of simple community structures, including diverse archaea, such as the phyla *Methanobacteriota*, *Thermoproteota*, and *Halobacteriota*. Asymptomatic individuals showed high participant-dependent intervariability in their urogenital archaeomes. The mean relative archaeal abundance was estimated to be 0.89%, and fluorescence in situ hybridisation micrographs provided evidence of archaeal cells in the human urogenital tract. In addition, the urogenital archaeome shared partial taxonomic compositional characteristics with those of the other body sites. In this study, *Methanobacteriota*, *Thermoproteota*, and *Halobacteriota* were suggested as inhabitants of the human urogenital tract, and a distinct human urogenital archaeome was characterised. These findings expand our knowledge of archaea-host associations in the human urogenital tract and may lead to novel insights into the role of archaea in urinary tract health.

## Introduction

The ‘sterile urine’ paradigm was rapidly outdated with the discovery of urogenital microbiota in both males and females by Nelson et al.^1^ and Siddiqui et al.^2^, respectively, followed by confirmation that most adults harbour bacterial communities in their lower urinary tracts^1–5^. Since its identification, the association between urogenital microbiota and human health has become a focus of research, and urogenital microbiota has been found to play a role in maintaining homeostasis in the urinary tract by modulating the immune response^6^. A relationship between the urogenital microbiota and urinary disorders has also been identified in patients with bladder cancer, urgency urinary incontinence, or chronic prostatitis/chronic pelvic pain syndrome, and an alteration in the urogenital microbiota in these patients has been revealed compared to that in those not afflicted with these disorders^7–9^.

The impact of human-associated microbiota on health has been demonstrated, with most researchers considering the connection between bacterial communities and human health. However, recent evidence has expanded this search and an exploration of the non-bacterial microorganisms inhabiting several parts of the human body and their role(s) in human health and diseases has been initiated. In particular, alteration of the archaeal communities is reported to be related to various disorders, even though these communities are only rarely found in human body parts, such as the gut, skin, and nasal cavity. The gut archaeome can differ considerably between humans with and without disorders; gut halophiles have been found to be significantly enriched in patients with colorectal cancer (CRC), while methanogens are depleted^10^. Skin archaeomes predominantly comprise *Thermoproteota* and *Methanobacteriota* and are known to be associated with both aging and dermatitis^11–13^. Both *Methanosphaera stadtmanae* and *Methanobrevibacter smithii* have been found to increase with age, and *Thaumarchaeota* has recently been associated with dermatitis^14^. The nasal cavity archaeome resembles that of the skin and is dominated by *Thermoproteota* (*Nitrososphaera*) and methanogenic *Methanobacteriota* (*Methanosphaera* and *Methanobrevibacter*). Methanogenic archaea in nasal cavity are correlated with refractory sinusitis^15^. As archaea are accounted to inhabit various sites in the human body and have a potential role in human health, it is important that novel archaeomes in the human body are investigated. Since the development of next-generation techniques, efforts to seek novel human-associated archaeomes began with an exploration of the archaeal signatures on human skin^12^; the existence of diverse archaeal community were found in the gastrointestinal tract (GIT), lung, nose^13^, nasal cavity, mouth, and appendix^11^. In addition, a diversity of haloarchaea was detected in the human gut in our previous research^16^. Nevertheless, only one attempt has been made to investigate the archaeal community in the urinary tract which revealed the presence of several phylotypes of methanogenic archaea in the postmenopausal urogenital microbiome^17^.

Most trials involving the relationship between the urogenital microbiome and host using culture-independent methods have investigated bacterial microbiota, with few focusing on non-bacterial members. Bacteriophage sequences have been found in the bacterial genome of the bladder, and a novel coliphage has been isolated from a bladder *Escherichia coli* strain. Variation in the phage populations of women with or without overactive bladder symptoms has been observed, suggesting the potential contribution of bacteriophages to urinary health^18^, while another study revealed the presence of diverse fungal community structures in the urogenital mycobiome of asymptomatic controls^19^, These results imply that other minor biological entities, such as archaea, could be present and even possibly thrive in the urogenital tract.

This study is an investigation of the archaeal community in the urogenital tracts (hereafter referred to as the urogenital archaeome, according to standard terminology suggested by Brubaker et al.^20^) of voided urine samples from asymptomatic Korean individuals using an archaea-specific metataxonomic approach. DNA-based approaches, including next-generation sequencing and quantitative PCR, were applied to uncover the previously unknown diversity and abundance of urogenital archaea. Fluorescence in situ hybridisation (FISH) was used to detect archaea in the urogenital tract. Non-methanogenic archaea, belonging to *Thermoproteota* and *Halobacteriota,* which had not been found previously in the urogenital tract, were observed. Overall, this study showed the existence and variety of urogenital archaea and calculate the quantitative differences of this microbiome from that of urogenital bacteria.

## Results

### Korean participants are all asymptomatic subjects

A total of 373 Korean participants were randomly collected to explore the urogenital archaeome of asymptomatic humans (females, n = 232; males, n = 141). No urinary tract infections (UTI) were observed during sample collection. Participants included 38% males and 62% females with an average age of 41.8 ± 14.4 and an average BMI of 22.4 ± 3.6. They were classified into archaea-positive and archaea-negative based on the results of archaeal 16S rRNA gene amplicon sequencing. The information on the gender, age, and BMI of each group is presented in Table 1.

**Table 1:** Baseline characteristics of participants stratified by presence of archaea.

Presence of archaea	Gender	Age	BMI
Archaea-positive	Total (n = 14)	33 ± 12.0*	22·6 ± 2·21
	Female (n = 10)	31 ± 13.2	22·0 ± 2·38
	Male (n = 4)	38 ± 6.4	24.0 ± 2.21
Archaea-negative	Total (n = 359)	41 ± 14.4*	23.4 ± 3.84
	Female (n = 222)	40 ± 13.9	22.4 ± 3.79
	Male (n = 137)	42 ± 15.1	24.9 ± 3.41

Data are expressed as the mean ± SD. Statistical analysis between archaea-positive and -negative groups was performed using Pearson’s chi-squared test (for the difference in gender) or Mann–Whitney *U*-test (for the difference in age and BMI). *P < 0.05.

### Detected archaeal sequences are not from environmental contaminant DNA

Since contamination with environmental DNA sequences derived from the reagents or kits used could cause bias in low-biomass microbiome studies with high PCR cycle numbers, the negative controls were deep-sequenced and analysed to confirm that the detected archaeal sequences were derived from the samples only. A total of 82,094 reads were obtained from the negative controls, with 1.83% of the reads (1505) remaining after denoising steps. Three short ASVs were obtained and could not be assigned to any archaeal taxa (see Supplementary Table S1 online) under taxonomic assignment using QIIME2. The BLAST results of these sequences showed no match with archaeal sequences but were instead associated with bacteria or phage sequences. Thus, the detected archaeal sequences in the samples were considered to originate from human urine specimens and not environmental contaminant.

### The urogenital archaeomes of asymptomatic human reveal low diversity with archaeal community structures that are highly dependent on the participant

**Figure 1:**
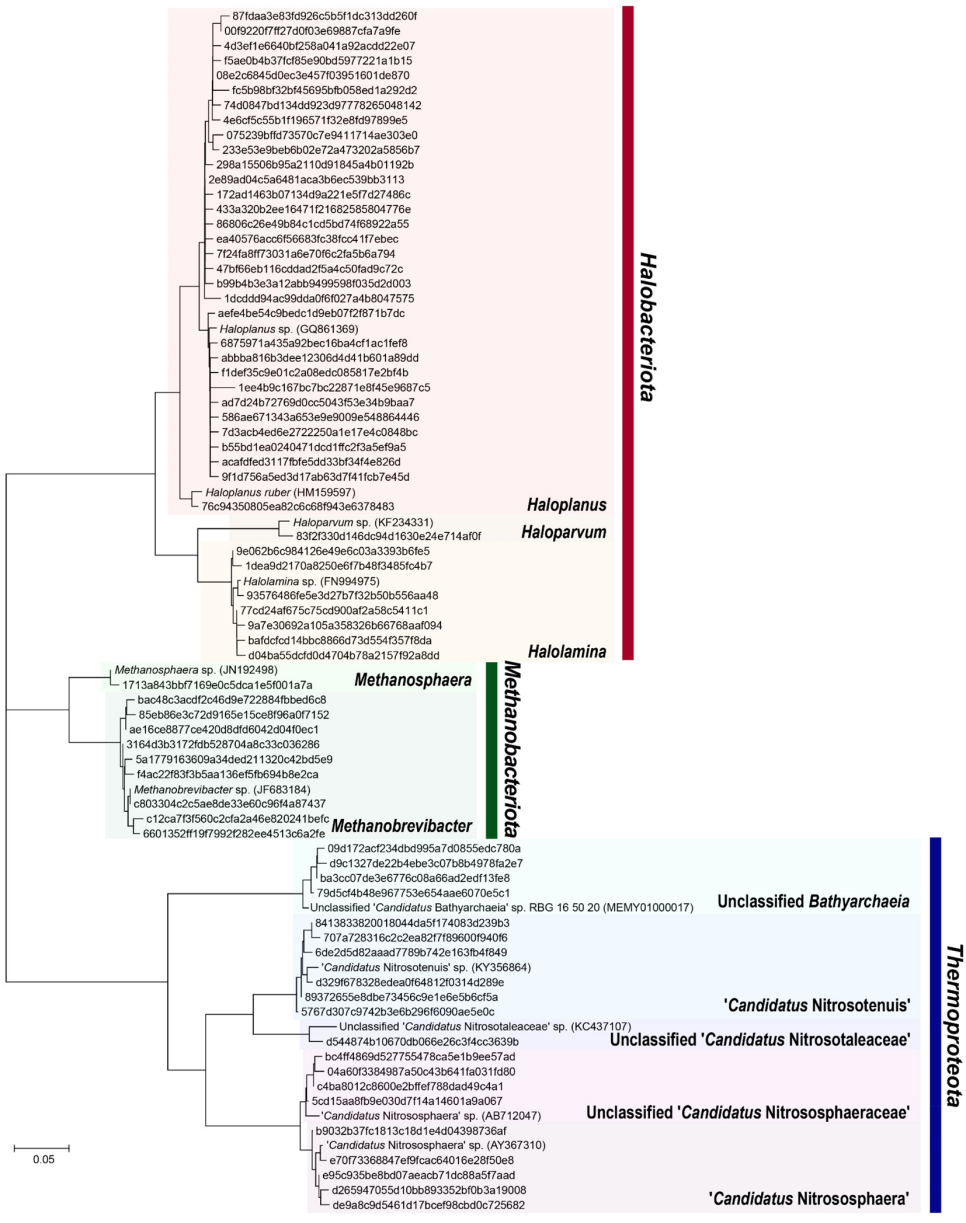
Phylogenetic tree based on 70 archaeal amplicon sequence variants (ASVs) from the urogenital tract of asymptomatic humans. The ASVs were assigned to the phyla *Halobacteriota* (red), *Methanobacteriota* (green), and *Thermoproteota* (blue).

**Figure 2:**
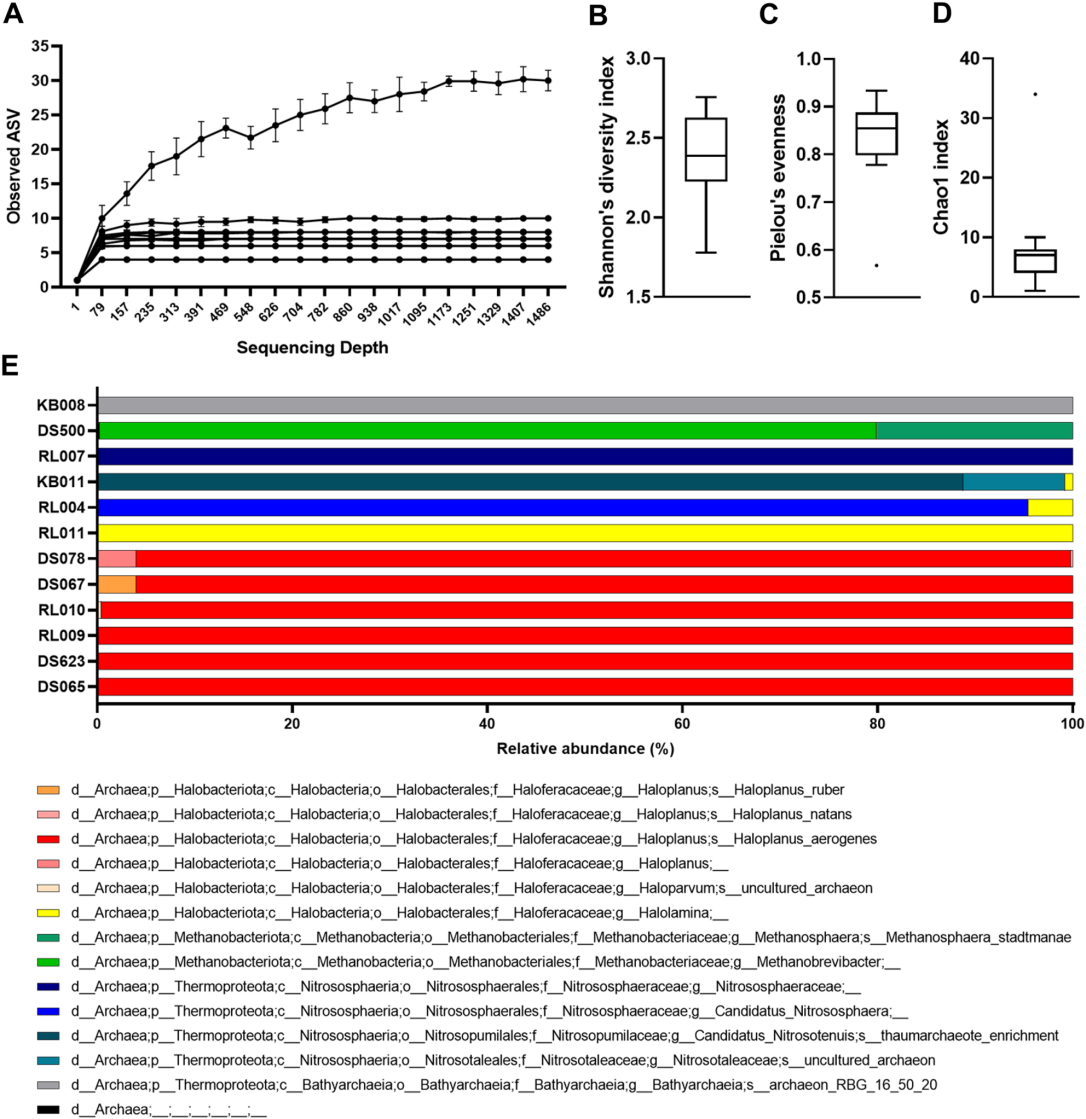
Diversity and community structure of the urogenital archaeome. (**A**) Rarefaction curve of the urogenital archaeome shows the level of saturation of ASV richness. (**B**) Shannon’s diversity, (**C**) Pielou’s evenness, and (**D**) Chao1 indices for the urogenital archaeome. (**E**) The 12 archaea-positive community structures show the relative abundances of taxa at the genus level. Each colour indicates different genus consisting of the assigned ASVs.

A urogenital archaeome was detected in 14 (3.75%) of the 373 asymptomatic participants, ten of whom were females (4.3%) and four were males (2.8%). Except for age (*P* = 0.0341), no significant differences were observed for any other characteristics, namely gender (*P* = 0.4678) and BMI (*P* = 0.5290), between archaea-positive and -negative groups (Table 1). The sequence length distribution of the raw FASTQ files for all samples was provided in Supplementary Table S2. The number of reads for archaea-positive samples after denoising was 9618 ± 20,845 (max = 80,457 and min = 399; Supplementary Table S3). Two male samples containing fewer than 1000 reads after denoising were discarded. The urogenital archaeome in 12 samples showed 70 representative ASVs (Fig. 1), and the rarefaction curves indicate saturation at the ASV level (Fig. 2A). The average of 8.75 ASVs per individual (max = 29 and min = 4) and low Shannon’s diversity and Chao1 indices (Fig. 2B–D) indicate that the community structure is low in richness and evenness but highly participant-dependent (Fig. 2E).

The ASVs in urogenital archaeome indicate the presence of various archaea, including *Methanobacteriota*, *Halobacteriota*, and *Thermoproteota* (see Supplementary Table S4 online). Methanogenic *Methanobacteriota* (nine ASVs), *M. stadtmanae* and *Methanobrevibacter* species, were detected in one female participants. Interestingly, although *Halobacteriota* had previously not been detected in the urogenital tract, the most prevalent ASVs (40 ASVs) belonged to the phylum *Halobacteriota,* and these *Halobacteriota*-assigned ASVs were observed in nine participants. The genera and species to which most ASVs were assigned were members of *Halobacteriota*, *Haloplanus* (32 ASVs) and *Haloplanus aerogenes* (18 ASVs), respectively. Other genera, such as *Halolamina* (seven ASVs) and *Haloparvum* (one ASV) were also detected. The sequence assigned to *Thermoproteota* (20 ASVs) was observed in four participants. Specifically, the sequences were assigned to ammonia-oxidizing archaea (order *Nitrosotaleales*, *Nitrososphaerales*, and *Nitrosopumilales*) and class *Bathyarchaeia*. The sequence similarity between urogenital archaeal ASVs belonging to the same genus was 94.4–99.3%, with ASVs belonging to the genus *Methanobrevibacter* showing 0.7–2.8% (1.7 ± 0.7%) sequence divergence and ASVs in the genera *Halolamina* and *Haloplanus* of the phylum *Halobacteriota* showing 97.9–99.3% (98.7 ± 0.5%) and 94.4–99.3% (97.3 ± 1.1%) sequence similarities, respectively. A pairwise sequence comparison of the ASVs in each genus of *Thermoproteota* revealed sequence similarities at 98.6–99.3% (98.9 ± 0.4%) in the unclassified *Nitrososphaeraceae*, 98.9–99.3% (98.9 ± 0.3%) in ‘*Candidatus* Nitrososphaera’, 97.9–99.3% (98.7 ± 0.6%) in ‘*Candidatus* Nitrosotenuis’, and 98.6–99.3% (99.0 ± 0.4%) in the unclassified *Bathyarchaeia* sp. Sequence similarity comparison between the urogenital archaeal ASVs and previously isolated type strains of valid taxa or strains of Candidatus taxa showed various values per genus, ranging from 87.5 to 100% (see Supplementary Table S5 online). *Haloparvum*-assigned ASVs showed the most divergent sequence similarities (87.5%) with the type strain of *Haloparvum alkalitolerans*, as compared to ASVs assigned to the other genera. Two ASVs assigned to the genus *Methanobrevibacter* revealed the most similar sequence identity (100%) with *M. smithii* and *Methanobrevibacter thaueri* type strains. The results obtained from deep sequencing suggest the possibility that diverse archaeal phylotype candidates exist in the human urogenital tract, which is significantly different from the methanogenic archaeal species detected previously.

### Archaea are minor residents in the human urogenital tract

**Figure 3:**
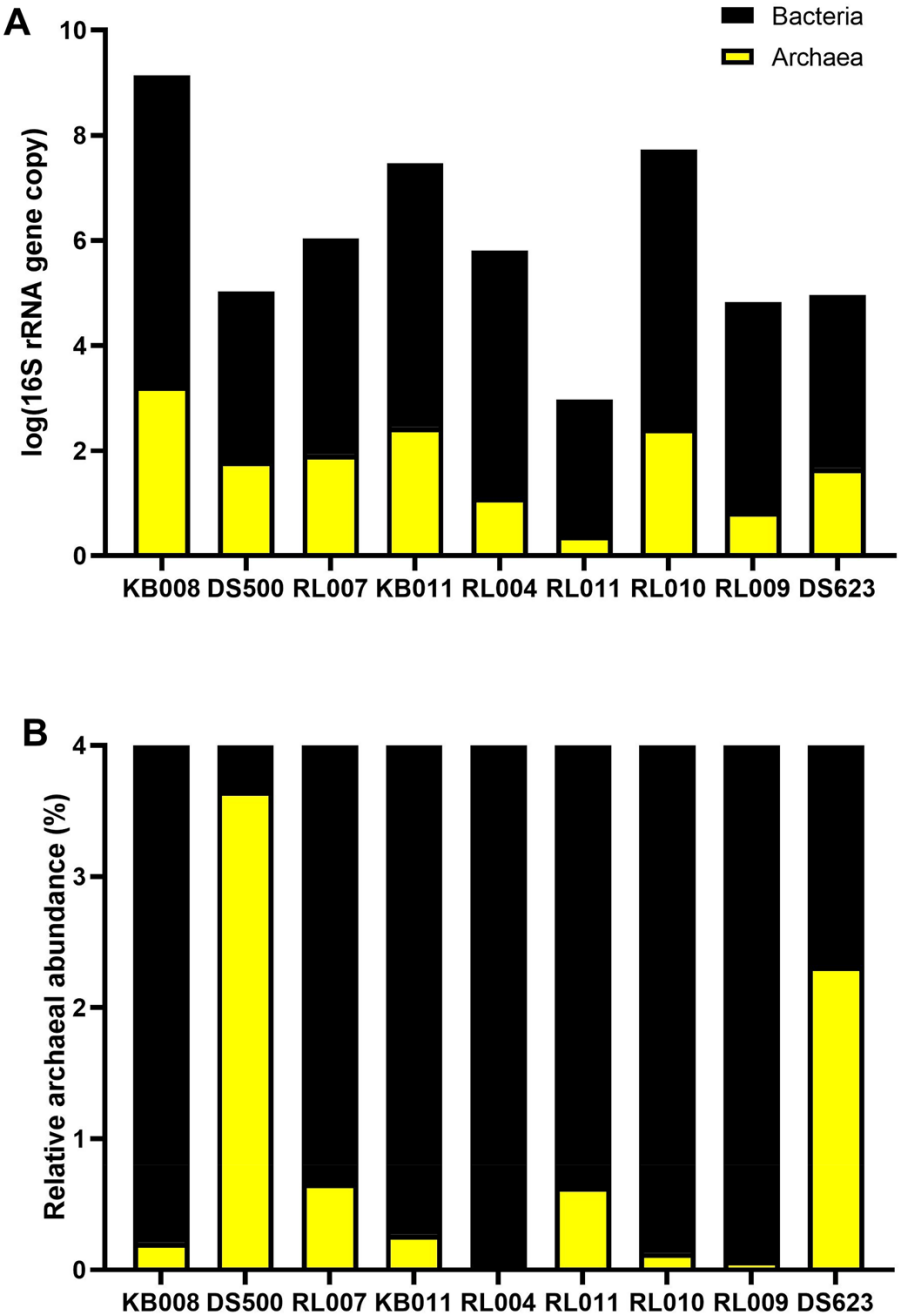
Quantification of human urogenital tract archaea. (**A**) The estimated logarithmic 16S rRNA gene copy numbers of archaea and bacteria. (**B**) Percent urogenital tract archaeal abundance per sum of abundances of archaea and bacteria.

Archaeal and bacterial abundances in the urogenital tract of archaea-positive asymptomatic humans were quantified using qPCR (Fig. 3). Standard curves previously obtained by Kim et al.^16^ were used for quantification analysis. The percent archaeal abundance was determined as the fraction of archaeal abundance over the sum of archaeal and bacterial abundance (Fig. 3B). High variability was revealed among participants in terms of the 16S rRNA gene copy numbers and the proportions of each in the urogenital tract of asymptomatic humans. The average bacterial 16S rRNA gene copies of 6.66 × 10^4^ per 100 ng DNA (ranging from 1.90 × 10^2^ to 4.06 × 10^5^) were 26- to 3900-fold that of archaeal gene copies (1.37 × 10^2^ ± 2.57 × 10^2^ per 100 ng DNA; Fig. 3A). The mean archaeal percent abundance was 0.89% (ranging from 0.03 to 3.65%), which revealed that urogenital archaea exist at a lower rate than urogenital bacteria. It is expected that further studies quantifying urogenital archaeal abundance proportions at the population level and a connected analysis of its abundance and clinical metadata might unveil the role and mechanism of archaea in relation to urinary health.

### Non-sequencing-based analysis reveals the presence of archaeal cells in the human urogenital tract

**Figure 4:**
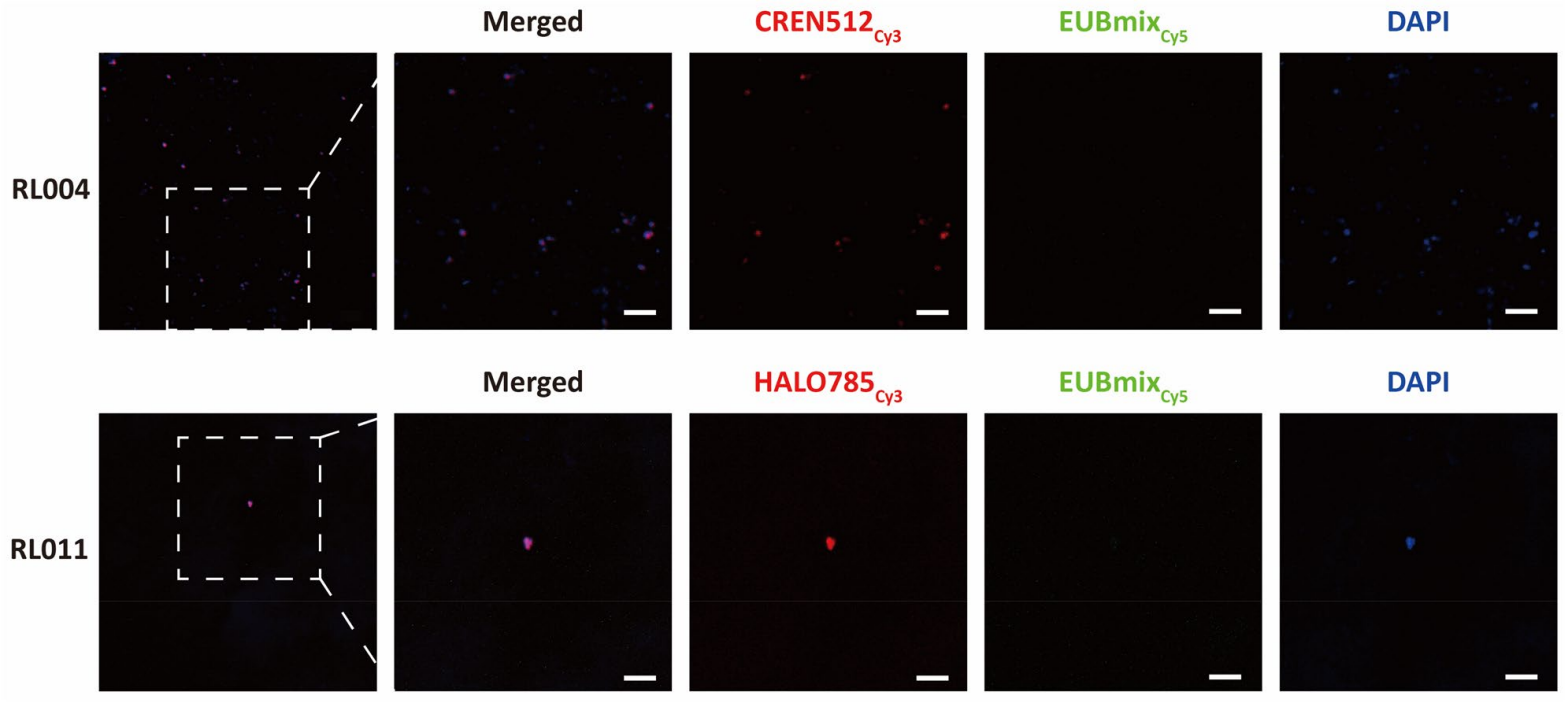
Detection of viable urogenital tract archaea in asymptomatic humans using fluorescence in situ hybridisation (FISH). The *Thermoproteota*- and *Halobacteriota*-rich urine specimens (RL004 and RL011, respectively) were subjected to the analysis. Probes CREN512_cy3_, HALO785_cy3_, and EUBmix_cy5_ were used to detect *Thermoproteota*, *Halobacteriota*, and bacteria, respectively. Scale bars correspond to 5 μm.

FISH analysis was conducted to determine the presence of urogenital tract archaea using a non-sequencing method. Neither *Halobacteriota* nor *Thermoproteota* have been detected previously in the urogenital tract. Two samples including either *Halobacteriota*- or *Thermoproteota*-related ASVs in the community profiles based on the 16S rRNA gene sequences (Fig. 4), were therefore selected to demonstrate the presence of these archaea via FISH analysis; both samples showed positive signals for *Halobacteriota* or *Thermoproteota* (Fig. 4). The overall results support the possible presence of non-methanogenic archaea in the urogenital tract.

### Urogenital archaeomes tend to cluster together and share taxonomic features with those of other human body sites

**Figure 5:**
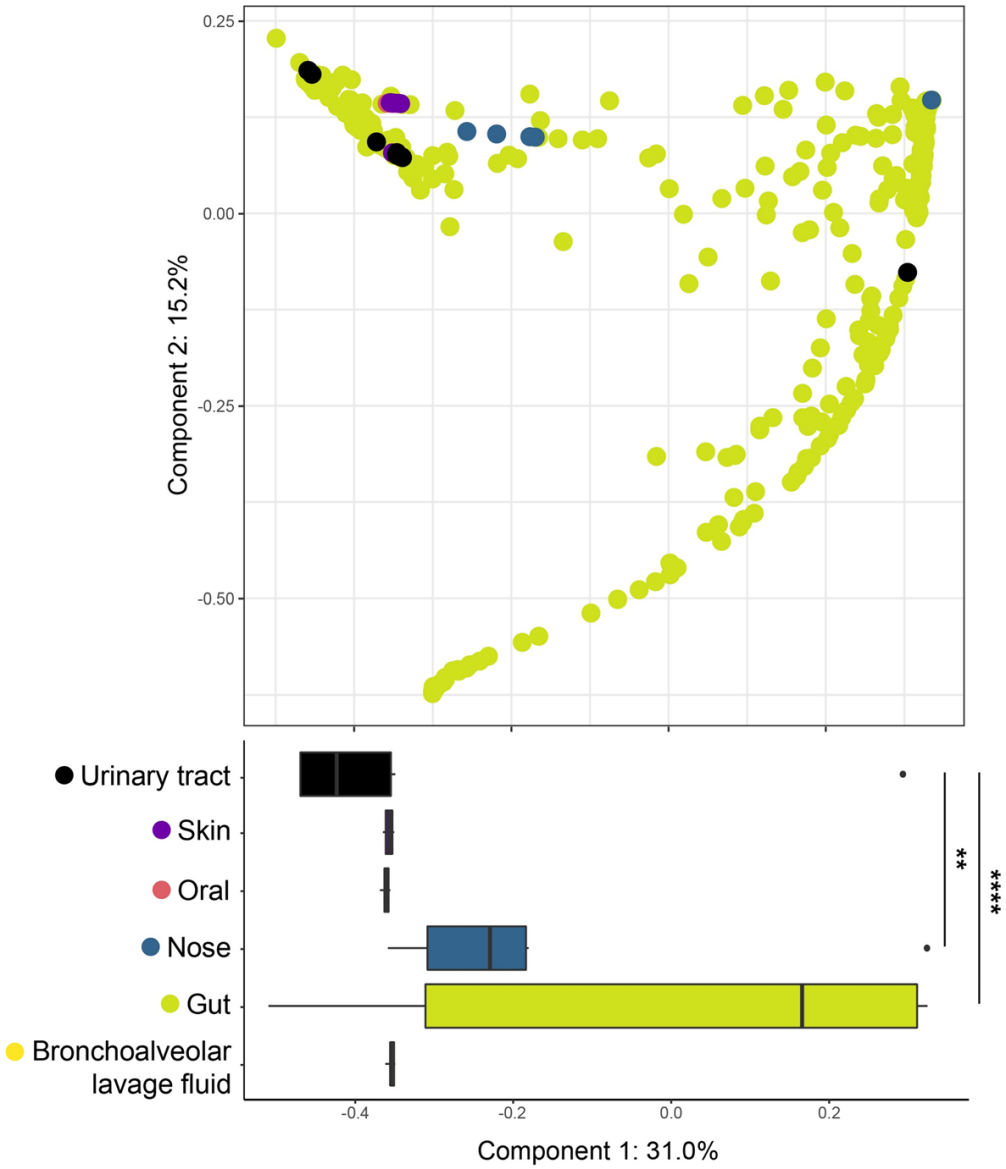
Comparison of archaeome of the urogenital tract and other human body sites. Principal coordinate analysis plot based on Bray–Curtis distance matrix of species level-relative abundances. Differences among all groups and between two groups were analysed using the Kruskal–Wallis (*P* < 0.0001) and Mann–Whitney *U*-test, respectively. The lines, boxes, and whiskers in the box plot diagrams represent the median, first, and third quartiles, and min-to-max distribution of replicate values, respectively.

The characteristics of the urogenital archaeome were indicated by metataxonomic and quantification data (Figs. 1, 2, 3); we next compared the urogenital archaeome with that of various human body sites such as gut, skin, oral, nose, and bronchoalveolar lavage fluid^11,13,16^ (GenBank accession number: PRJNA522626, PRJEB19529, and PRJEB27023). A principal coordinate analysis (PCoA) plot was generated using relative abundance at the species level for the archaeome at each body site based on the Bray–Curtis distance (Fig. 5). The boxplot in Fig. 5 indicates PC1 values of archaeome in various human body sites and significance when comparing PC1 values of urogenital archaeome and other body sites. The archaeome in urogenital tract could be differentiated from that in the gut (*P* < 0.0001) and nose (*P* < 0.01), respectively. Except for one urogenital archaeome that harboured unclassified *Bathyarchaeia* sp., most urogenital archaeomes were closely positioned. The gut archaeome was distributed in a horseshoe-shaped ordination and overlapped with the most archaeomes at other body sites. The archaeomes in the nose were generally located close together; however, the nose archaeome of one participant was positioned distantly from the others. The urogenital archaeome forms a small cluster that overlaps the PCoA plot where the oral, skin, and bronchoalveolar lavage fluid archaeomes are found. These results indicate that the urogenital archaeome shares some taxonomic compositional characteristics with that of other body sites. The urogenital archaeome, which showed individual participant-dependent high intervariability in archaeal community structures (Fig. 2D) and a quantification result (Fig. 3), revealed one mild cluster. Collectively, we suggest the possibility that the urogenital archaeome consists of urogenital tract-specific archaeal taxa that resemble archaeomes from other human body niches; exploration of this concept in further studies with a larger number of human populations is required for a better understanding.

## Discussion

This study investigated urogenital archaeal profiling based on 16S rRNA gene amplicon sequencing. To the best of our knowledge, no previous studies have revealed the presence of urogenital *Thermoproteota* and *Halobacteriota* using both sequencing and non-sequencing-based approaches. The urogenital tract archaeome showed distinct characteristics compared to those at other sites in the human body. The urogenital tract showed one of the simplest archaeal profiles observed as compared to those in the GIT, skin, and nose^13,16^, and formed a weak cluster among the archaeomes. Their occupancy ratio was only 0.80%, which is much smaller than that of the urogenital tract bacteria. Previous studies have shown the archaeal proportion was lower than bacteria in other human body sites, such as Korean gut with a mean archaeal proportion 10.24%^16^ and the human front torso with mean proportions 0.60%^21^ or 1.942%^12^. A direct comparison between urogenital and skin archaeal abundances is not possible due to differences in data collection methods, but it can be inferred that the ratio of archaea in the urogenital tract and skin is not similar to that in the gut, based on the data of the previous studies.

Despite the low abundance of urogenital tract archaea, the presence of these microorganisms may affect human health. One of the rare taxa in the human body, *Actinomycetota*, is described as a keystone taxon in the human gastrointestinal tract because of its strong influence and numerous linked interactions in the microbiome, with intimate interactions that are not proportional to the low abundance observed^22^. Changes in its abundance have been reported to be related to several diseases such as obesity^23^ and inflammatory bowel disease^24,25^. Studies on the effect that a low abundance of gut bacteria has on the microbial structure and gene expression in the host have suggested that low abundance bacteria can be potent drivers of inflammatory immune responses^26^. Based on these studies, inconspicuous archaea in the urogenital tract may be keystone microorganisms to affect the diversity of co-existing microorganisms and the host immune system. As mentioned above, archaea play important roles in human health. The well-known human-associated archaea, the methanogenic archaea, have been reported to be associated with UTIs in the human urogenital tract. According to Grine et al.^27^, 54% of patients harbouring *M. smithii* are diagnosed with UTIs, and *M. smithii* could be a player in community-acquired UTIs that are linked with enteric bacteria. In this context, further studies that explore the association of *Thermoproteota* and *Halobacteriota* with urogenital tract health or diseases might be valuable for elucidating the effect of archaea on human urogenital tract health.

Archaea in the human gut are also associated with human diseases such as inflammatory bowel disease (IBD) and colorectal cancer (CRC). A decrease in the abundance of *M. smithii* has been observed in IBD patients as compared to healthy controls^28^ and a significant enrichment of haloarchaea with a depletion in the methanogenic archaea have been observed in CRC patients. The abundance of nine archaeal species has been suggested as markers for CRC diagnosis^10^. The relevance of archaea to oral health or skin disease has also been reported by Horz and Conrads^29^ and Probst et al.^21^ The relative abundance of *Methanobrevibacter oralis* increased as the severity of the oral disease increased but was not detected in the mouths of healthy individuals. An increase in skin archaeal abundance was detected in human participants younger than 12 and older than 60 years old. Since the skin moisture content decreases as archaeal abundance increases, it has been suggested that archaea are associated with age and a reduction in the skin sebum and lipid content^21^. Little is known about the mechanisms by which archaea contribute to human health; however, several evidences have been reported in association with the immune system. Methanogenic archaea can drive the production of pro-inflammatory cytokines (Th-1, Th-2, and Th-17), modulate the release of antimicrobial peptides, and activate TLR8-dependent NLRP3 inflammasomes^30^. One member of the haloarchaea, *Halobacterium salinarum*, is known to be able to induce CD8^+^ T cell immunity^31^. Taken together, even small abundances of microbes could play keystone roles in disease or immunity, and even archaea that account for minor proportions of the human microbiome have continuously been reported to be associated with aspects of human health. Bacteria, also found in the urobiome, are associated with urinary tract health^32^, and urogenital archaea, even in small proportions, may also be involved in urinary diseases or contributing to the homeostasis of the urinary system, for people harbouring archaea in their urogenital tract.

The human urogenital tract is not a favourable environment for microbial growth. Urine is a hypertonic environment with a low pH (less than 6.0), low levels of oxygen, and a high concentration of urea^33,34^. However, archaea have the potential to survive in harsh environments. Archaea can live in a wide range of habitats, ranging from extreme (ex. salt lakes, guts, and hydrothermal vents) to mild environmental conditions. Most archaea use osmoadaptive strategies to increase the intracellular concentrations of inorganic cations (ex. K^+^)^35^, therefore urogenital archaea may adapt to the hypertonic condition of urine using this strategy. According to Bouatra et al.^36^, various carbohydrates, amino acids, organic acids, and inorganic compounds are present in human urine, and archaea have the potential to be associated with urine metabolite production by using various urinary compounds. Urine contains compounds that act as alternative electron acceptors for most anaerobic haloarchaea (i.e. nitrate, trimethylamine-N-oxide, and fumarate)^37^. *Bathyarchaeia* may contribute to acetogenesis in the urine via the production of acetate from various urinary organic acids^38^. These assumptions would be further validated using metagenomic and metatranscriptomic approaches. This effort may contribute to understanding the interaction between the human urogenital tract and urogenital archaea and the mechanisms by which urogenital archaea affect human health.

The origin of the urogenital microbiota has not yet been unveiled; however, several assumptions have been introduced from the GIT, vagina, and oral cavity, and the interaction between the urogenital tract and these environments in several studies. Bacterial species previously isolated from the gut and vagina have been isolated from human urine specimens^39^ and bacterial genera detected in the gut, vagina, and oral cavity have also been detected in the urogenital microbiota^40^. Collectively, it may be assumed that latent sources of urogenital microbiota are present in the GIT, vagina, and oral cavity, and urogenital tract archaea may have originated from these sources in the same manner. The phyla of archaea detected in this study, *Halobacteriota* and *Thermoproteota*, have been found in the human GIT, and the methanogenic archaea, *Methanobrevibacter* and *Methanosphaera*, were the first archaea isolated from human and have been detected in the human GIT and vagina^41^. However, although the provenance of the urogenital tract archaea was assumed to be from such a source, the distinctiveness of the microbial profiles in the urogenital tract as compared to those found in the GIT is obvious (Figs. 2D, 5). One previous study^4^ also suggested a difference between the microbial profiles of female urinary bladder and the vagina. In addition, the microbiota of the human gut and bladder harbour different characteristics in terms of community profile^42^. Collectively, as the precise origin of urogenital tract microbes has not been fully investigated, further studies are required to elucidate the origin of urogenital archaea using microbial culturomics to isolate intact viable archaea.

We investigated the existence, diversity, and abundance of archaeomes in the asymptomatic human urogenital tract via archaeal metataxonomics and the presence of archaeal cells in the urine using FISH. The human urogenital archaeome showed low richness, high intervariability, and distinct characteristics compared to the archaeomes of other human body sites. However, the overrepresentation of short reads is a major limitation of this study as it led to the removal of the majority of reads in the dataset during quality control (Supplementary Table S2). Further studies comparing the urogenital archaeome of patients with UTIs, the bladder colonization mechanism of archaea, their interaction with other urogenital tract microbes and archaea, and the connection between archaea and various clinical metadata would be helpful to investigate the effect of archaea on urinary tract health. In addition, comparing the archaeome from catheterised and voided urine specimens and the archaeome of the urinary tract and other human body sites could clarify more characteristics of the urinary tract archaeome.

## Conclusion

We investigated the urogenital tract archaeome profiles of asymptomatic human participants using high-throughput amplicon sequencing. We confirmed the existence of diverse archaea that have not been detected previously using both high-throughput sequencing and non-sequencing-based methods. The urogenital archaeomes of asymptomatic humans revealed simple community structures. In addition, we showed that the abundance of urogenital tract archaea was much lower than that of the bacteria. Therefore, we can conclude that various archaea could be minor inhabitants of the urogenital tract of asymptomatic humans and may interact with urinary tract health.

## Materials and methods

### Sample collection

A total of 373 human participants without urinary tract infection and living in Korea were recruited from the Naju Korean Medicine Hospital of Dongshin University. The participants fasted for 12 h before urine sample collection. The voided urine samples were collected in a plastic tube that was kept on ice for transport to the laboratory. Before storage at − 80 °C in a freezer, 1 ml of 10% solution (100 ml isopropanol and 10 g thymol) was added per 30 ml of the urine samples for urine metabolome analysis designed for a separate study. The frozen urine samples were thawed on ice and aliquoted for microbial analysis including DNA extraction and FISH assay. The study was conducted in accordance with the relevant guidelines, regulations, and principles for human research. All participants provided written informed consent. The study protocol was approved by the institutional review board (IRB) of Dongshin University (IRB No.DSMOH19-1).

### DNA extraction from voided urine specimens

Genomic DNA was extracted from the urine specimens according to the protocols by Zainabadi et al.^43^ using the extraction procedures described below. A lysis buffer for the DNA extraction was prepared using 3 M guanidine thiocyanate, 33% isopropanol, 4% Triton X-100, 50 mM EDTA, 20 mM Tris HCl (pH 7.4), and 0.5% 2-mercaptoethanol. Washing buffer 1 comprised diluted lysis buffer (w/o 2-mercaptoethanol) with DW in a 1:1 ratio and washing buffer 2 consisted of 25% ethanol, 25% isopropanol, 100 mM NaCl, and 10 mM Tris HCl (pH 7.4). Silicon dioxide (2.5 g) was mixed with 20 ml of lysis buffer after size selection and used as a binding solution. Urine (10–20 ml volume) was placed in a 50 ml Falcon tube and mixed vigorously with the same volume of lysis buffer and 2% of binding solution. The resulting sample was then centrifuged (1640×*g*) for 1 min and the supernatant was discarded. A 2% sample volume of washing buffer 1 was added to the pellet and the mixture was vortexed, centrifuged (2250×*g*, 1 min), and the supernatant discarded. The washing step was performed twice before the same volume of washing buffer 2 was added to the pellet. The mixture was vortexed, centrifuged (2250×*g*, 2 min), and the supernatant discarded. The pellet was dried at 60 °C for 10 min before the DNA was eluted with 100 μl of DNase-free water and the mixture was incubated for 1 min. The eluted DNA was separated from the silicon dioxide by centrifugation at 2250×*g* for 2 min and stored at − 20 °C. Blank DNA extraction controls were included following all steps of the process and served as negative controls.

### Archaeal 16S rRNA gene amplification

The archaeal 16S rRNA gene was amplified using nested PCR to screen archaea-positive samples^13^. Primer pairs were used as described previously^16^. Briefly, the S-D-Arch-0344-a-S-20 and S-D-Arch-0911-a-A-20 primer pairs were used for primary PCR, and the S-D-Arch-0349-a-S-17 and S-D-Arch-0519-a-A-16 primer pairs with Illumina overhang adapter sequences for secondary PCR. Premix products from the Maxime™ PCR PreMix (i-StarTaq; iNtRON Biotechnology) and the MG 2X PCR mastermix (MGmed) were used for primary and secondary PCR, respectively. The first amplification reaction was performed for 10 cycles of 30 s at 96 °C, 30 s at 60 °C, and 1 min at 72 °C; 20 cycles of 25 s at 94 °C, 30 s at 60 °C, and 1 min at 72 °C with initial denaturation performed for 2 min at 95 °C and a final extension lasting 10 min at 72 °C. The second PCR was performed for 30 cycles of 40 s at 95 °C, 2 min at 63 °C, and 1 min at 72 °C, with an initial denaturation of 5 min at 95 °C and a final extension lasting 10 min at 72 °C. The template DNA for the second PCR was prepared by purifying the first PCR amplicons using the x-tracta™ Gel Extractor (Promega) and Qiaquick Gel Extraction Kit (QIAGEN). Ten replicates of the amplified PCR products were pooled for downstream analysis.

### Library preparation and 16S rRNA gene amplicon sequencing

Libraries for amplicon sequencing were prepared using the Herculase II Fusion DNA Polymerase Nextera XT Index Kit V2 (Illumina), following the manufacturer’s instructions. The DNA molecules were ligated with indices and the target size DNA band was extracted, as described above. DNA quantification and library size verification were performed using the PicoGreen and 4200 TapeStation system (Agilent Technologies), respectively. Samples for sequencing were selected based on the presence of a peak at the target size. Fourteen of the 373 samples were thus sequenced using the Illumina Miseq platform. The samples with blank DNA extraction, which were subjected to the same procedures were pooled and also sequenced as a negative control to assess the possibility of contamination.

### Sequence data processing and analysis

Raw sequence data were converted into FASTQ files, and the FASTQ reads were adapter-trimmed and demultiplexed using the conversion software bcl2fastq v.2.20.0. The sequences were processed and analysed using QIIME2 v.2021.4^44^ as described below. The reads were imported into QIIME2 and the primer sequences trimmed using the q2-cutadapt plugin^45^. The DADA2 pipeline^46^ was used for sequence filtering, dereplication, denoising, chimeric sequence removal and merging paired-end reads. Taxonomic assignment was performed using the classify-sklearn method in the feature-classifier plugin with a pre-trained naïve Bayes classifier that was trained on the SILVA database SSU v.138.1 Ref NR 99^47^. Unassigned amplicon sequence variant (ASV) sequences from the negative controls were searched against the nr/nt database using BLASTn from the National Center for Biotechnology Information (NCBI)^48^. ASVs assigned to eukaryote (*Eukaryota*) and singletons were removed before further analysis. The resulting urogenital archaeal 16S rRNA gene sequences were compared with nine reference sequences from the SILVA database. Sequences were aligned using the neighbor-joining algorithm^49^ with Clustal W^50^ in BioEdit software (v.7.0.5.3). A phylogenetic tree was constructed with these aligned sequences using 1000 bootstrap replications in MEGA X software^51^. Evolutionary distances were calculated using the Kimura 2-parameter model^52^. The sequence similarity between urogenital archaeal ASVs and/or type strains of each genus was calculated using Clustal W^50^. The 16S rRNA gene sequences describing each type strain were obtained from the NCBI GenBank database^53^. Comparison of the archaeome in the urogenital tract and other human body sites at the species level was performed using QIIME2 and the R package *vegan*. Archaeal sequences from other parts of the human body in previous studies^13,16^ were obtained from the Sequence Read Archive at the NCBI^54^. For the comparative analysis, 381 faecal samples from the study by Kim et al.^16^ and 77 GIT samples, two nose samples, six skin samples, and 36 bronchoalveolar samples from the study by Koskinen et al.^13^, were included. In the study by Kim et al.^16^, the participants who had not consumed any antibiotics in the three months prior to the study and had no history of major gastrointestinal diseases, were included. A total of 723,741 ± 557,005 sequence reads was obtained per sample, using the HiSeq X platform. In the study by Koskinen et al.^13^, the volunteers to collect samples from GIT, nose, and skin, and the patients who had been non-neutropenic intubated and mechanically ventilated to collect bronchoalveolar sample, were recruited. The obtained sequence reads were 72,262 ± 72,968 per sample using the Miseq platform. Samples that did not exceed 1000 sequence reads after quality control using the DADA2 pipeline were excluded from the analysis.

### Archaeal abundance estimation using quantitative PCR

To compare archaeal 16S rRNA gene copy numbers with those of bacteria, real-time quantitative PCR (qPCR) was performed according to Kim et al.^16^ using the CFX96 real-time PCR detection system (Bio-Rad). The primers used in the archaeal secondary PCR (without adapter sequences) and the primer pairs Bac1055YF and Bac1392R^55^ were used for archaeal and bacterial sequence amplification, respectively. DNA samples (2 ng, n = 9) prepared for each reaction comprised a 20 μl reaction volume with 10 μl TOPreal qPCR 2X premix (Enzynomics) and 300 nM of each forward or reverse primer. A non-template control was included, and all samples and controls run in triplicate. The estimated archaeal and bacterial 16S rRNA gene copy numbers were adjusted using the suggested average number of archaeal and bacterial 16S rRNA genes per genome (1.7 and 5.0, respectively)^56^.

### FISH assay

The oligonucleotide probes were conjugated at the 5′-end with Cy3 to target *Halobacteriota* or *Thermoproteota*, Cy5 to target bacteria, and FAM for a non-targeting probe. Bacteria, *Halobacteriota*, and *Thermoproteota* were assessed by multicolour analysis using the probes EUB338mix (EUB338, EUB338-II, and EUB338-III)^57^, HALO775^16^, and CREN512^58^, respectively. The in silico probe specificities of EUB338mix, HALO775 and CREN512 for bacteria, *Halobacteriota* and *Thermoproteota*, respectively, were confirmed using ProbeBase (https://probebase.csb.univie.ac.at/). The binding specificities were also experimentally tested (Supplementary Figure S1). The NONEUB probe^59^ was used as a negative control in all assessments. Cells in the urine samples were detected using fluorescence in situ hybridisation^60^ with minor modifications. Briefly, cell fixation was performed by adding 3 volumes of 4% paraformaldehyde to each urine specimen, which was followed by incubation at 4 °C for 4 h. The samples were then centrifuged at 6000×*g* for 5 min at 4 °C and the resulting pellets washed three times with PBS, as described above. The pellets were suspended in PBS and 100% ethanol of the starting volume of each sample. The suspension was seeded on a glass slide (Fisherbrand Superfrost Plus Microscope Slides; Thermo Fisher Scientific) and dried at 46 °C for 30 min. Cells were dehydrated in 50%, 80%, and 100% ethanol for 3 min each before hybridisation was performed for 2 h at 46 °C using a 20% formamide hybridisation buffer. To prevent non-specific binding of the probe molecules, the slide was rinsed off with pre-warmed washing buffer and incubated at 48 °C for 20 min. Cells were then stained with DAPI after the slide was completely dried. A confocal microscope (LSM710; Carl Zeiss, Oberkochen, Germany) at ×1000 magnification and the associated software (ZEN v.3.1, blue edition, Carl Zeiss) were used.

### Statistical analysis

Significant differences between groups were analysed using the Mann–Whitney *U*-test or Pearson’s chi-squared test in R. The statistical significance of the observed variations was tested using PERMANOVA with the adonis2 function in the R package *vegan*^61^.

## Supplementary Information

Supplementary Information.

## Data Availability

Raw sequence data were deposited in the NCBI Sequence Read Archive under BioProject Number PRJNA837385.
