# Supplementary material for: In-depth metataxonomic investigation reveals low richness, high intervariability, and diverse phylotype candidates of archaea in the human urogenital tract
Source: Sci Rep. 2023 Jul 20;13:11746. doi: 10.1038/s41598-023-38710-9 (PMC10359320; doi:10.1038/s41598-023-38710-9)
Supplement: Supplementary file 1 — Supplementary Information. [file 41598_2023_38710_MOESM1_ESM.pdf]

Supplementary information

In-Depth Metataxonomic Investigation Reveals Low Richness, High Intervariability, and Diverse Archaeal Phylotypes Candidates in the Human Urogenital Tract

Yeon Bee Kim[a,b#], Tae Woong Whon[a#], Joon Yong Kim[c], Juseok Kim[c], Yujin Kim[a], Se Hee Lee[a], Seong-Eun Park[d], Eun-Ju Kim[d], Hong-Seok Son[d*] and Seong Woon Roh[c*]

[a]Kimchi Functionality Research Group, World Institute of Kimchi, Gwangju 61755, Republic of Korea

[b]Department of Agricultural Biotechnology, Center for Food and Bioconvergence, Research Institute for Agricultural and Life Sciences, Seoul National University, Seoul, Republic of Korea

[c]Microbiome Research Team, LISCure Biosciences Inc, Gyeonggi-do 13486, Republic of Korea

[d]Department of Biotechnology, College of Life Sciences and Biotechnology, Korea University, Seoul 02841, Republic of Korea

[#]These authors contributed equally.

[*]Corresponding authors:

Hong-Seok Son

Department of Biotechnology, College of Life Sciences and Biotechnology, Korea University, Seoul 02841, Republic of Korea. Telephone: +82 2 3290 3053; E-mail: sonhs@korea.ac.kr

Seong Woon Roh

Microbiome Research Team, LISCure Biosciences Inc, Gyeonggi-do 13486, Republic of Korea. Telephone: +82 31 706 1712; E-mail: seong18@gmail.com

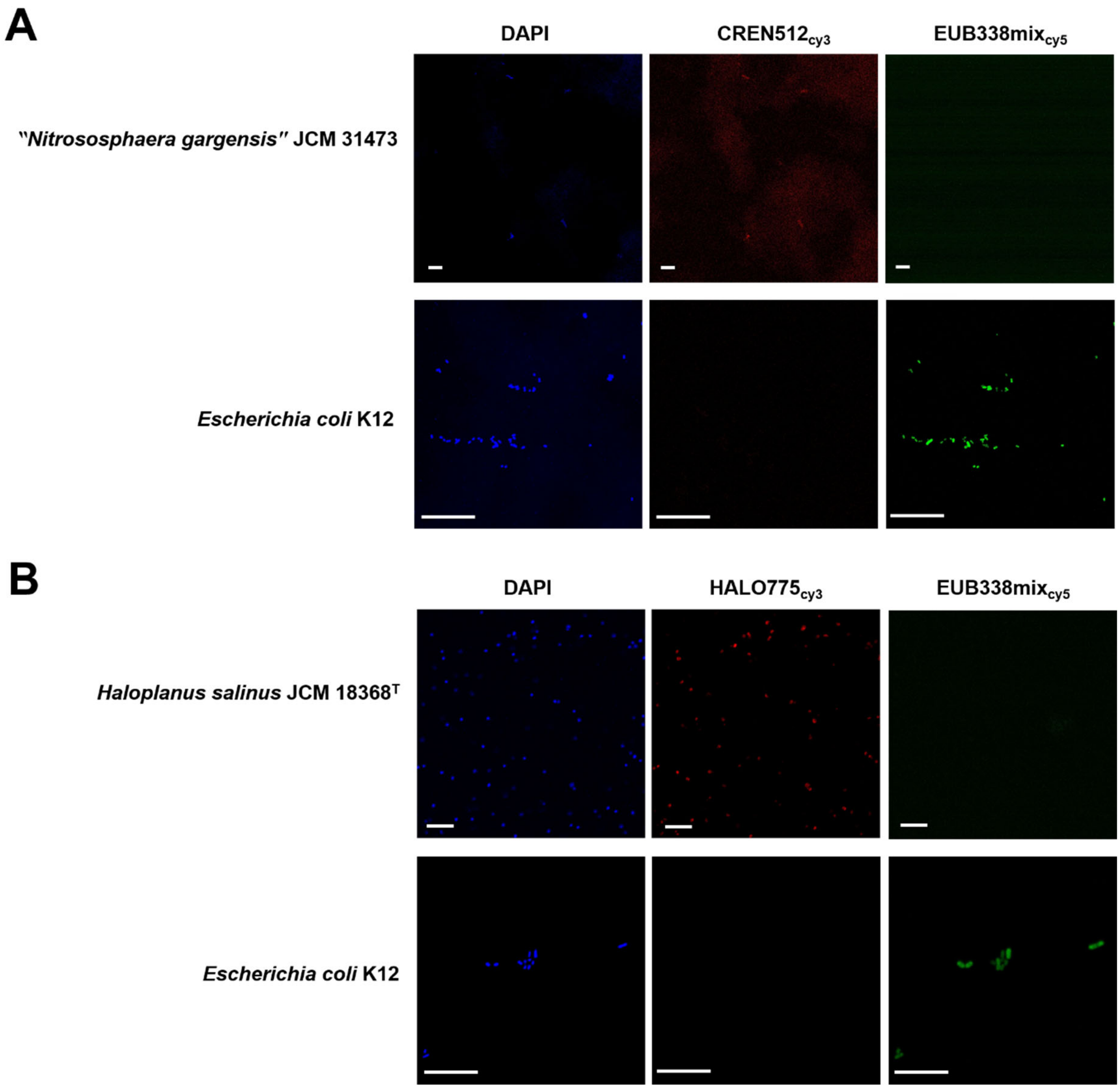

Supplementary Figure S1. Confirmation of binding specificity of EUB338mix$_{cy5}$ (green) (**A**,**B**) CREN512$_{cy3}$ (red) (**A**) and HALO775$_{cy3}$ (red) (**B**) probes by fluorescence in situ hybridization (FISH). The binding specificity of the probes to strains *Escherichia coli* K12, "*Nitrososphaera gargensis*" JCM 31473, and *Haloplanus salinus* JCM 18368^{T} was tested. A counterstain was performed using DNA staining solution (4′,6-diamidino-2-phenylindole, DAPI, blue). Scale bars corresponded to 10μm.

Supplementary Table S1. The sequences and assigned taxa of the predicted ASVs from sequences of negative controls

ASV ID	Sequence	Assigned taxon
8f00bd7453e402bc7931d2d9848c34fa	GTGCACCAGTCGCCGCGGTAA	Unassigned
7cf01598c20fa34558b01e9686d3cbfe	GTGCACCAGCCGCCGCGGTAA	Unassigned
b2638a2f40444ecae49f36091ec10686	GTGCAGCAGTCGCGGTAACTGTCTCTTATACACATCTCCGAGCCCACAGCCGCCGCGGTAA	Unassigned

Supplementary Table S2. The sequence length distribution of the raw FASTQ files for all archaea-positive samples

Sample	0-9	10-19	20-29	30-39	40-49	50-59	60-69	70-79	80-89	90-99	100-109	110-119	120-129	130-139	140-149	150-159	160-169	170-179	180-189	190-199	200-209	210-219	220-229	230-239	240-249	250-259	260-269	270-279	280-289	290-299	300-302
DS065_1	3	39,819	88	5	3	8	318	0	0	0	1	0	0	0	15,956	1	1	1	0	1	0	0	0	0	0	0	0	0	0	2	326
DS065_2	2	40,241	88	5	3	9	166	1	0	0	0	1	0	0	15,965	1	1	1	0	0	0	0	0	0	0	0	0	0	0	0	49
DS067_1	1	22,187	59	0	1	6	27	0	0	0	0	1	0	1	8,523	0	2	0	0	0	0	0	0	0	0	0	0	0	0	2	236
DS067_2	1	22,379	59	0	1	8	34	0	0	0	0	0	0	1	8,530	0	2	0	0	0	0	0	0	0	0	0	0	0	0	0	31
DS078_1	5	73,232	256	4	4	4	18	0	0	1	0	3	0	38	306,879	8	6	3	1	1	0	0	0	0	0	0	0	0	0	4	959
DS078_2	6	73,844	261	4	3	2	14	1	0	1	0	3	0	39	307,104	8	6	3	0	1	0	0	0	0	0	0	0	0	0	2	124
DS500_1	1	18,783	91	381	8	63	353	100	4	18	8	40	22	43	48,089	9	63	11	25	7	8	0	0	0	0	0	0	0	0	2	192
DS500_2	0	19,166	91	389	8	62	61	144	4	18	8	41	22	43	48,117	9	63	10	25	5	9	0	0	0	0	0	0	0	0	0	26
DS623_1	2	45,397	122	127	205	259	630	21	30	7	15	4	1	1	2,638	8	25	16	4	1	0	0	0	0	0	0	0	0	0	1	588
DS623_2	2	45,589	120	130	213	432	744	29	42	31	13	4	0	1	2,643	8	26	16	4	0	0	0	0	0	0	0	0	0	0	0	55
KB001_1	2	44,340	148	102	163	223	777	15	31	15	19	5	0	2	228	2	3	0	0	2	0	1	0	0	0	0	0	0	0	0	559
KB001_2	1	44,447	146	102	174	387	925	32	53	40	21	7	0	1	232	2	3	0	0	1	0	0	0	0	0	0	0	0	0	0	63
KB008_1	4	32,426	102	88	143	152	522	7	21	6	18	3	8	4	3,618	19	34	18	5	5	2	0	0	0	0	0	0	0	0	0	435
KB008_2	3	32,591	95	89	145	269	593	16	36	20	11	6	8	4	3,627	18	34	18	3	5	2	0	0	0	0	0	0	0	0	0	47
KB011_1	2	40,679	160	81	162	138	385	12	15	5	10	2	0	0	2,987	7	25	10	4	1	0	1	0	0	0	0	0	0	0	0	454
KB011_2	2	40,792	153	80	172	241	525	18	32	23	12	3	1	0	2,986	7	25	10	3	3	0	0	0	0	0	0	0	0	0	0	52
KB012_1	3	65,359	243	161	238	314	982	22	27	21	42	6	0	0	419	3	5	2	1	1	0	0	0	0	0	0	0	0	0	1	952
KB012_2	1	65,571	247	167	257	651	1,204	36	55	62	32	6	0	0	424	3	4	2	0	0	0	0	0	0	0	0	0	0	0	0	80
RL004_1	5	38,349	97	86	165	128	688	13	17	6	9	6	1	3	2,785	14	29	12	9	4	1	1	0	0	0	0	0	0	0	0	605
RL004_2	4	38,781	96	83	181	207	671	22	28	25	4	8	1	5	2,791	14	32	13	6	5	1	0	0	0	0	0	0	0	0	0	55
RL007_1	6	51,989	149	112	191	200	651	17	20	6	22	6	0	4	5,288	25	49	27	15	1	0	0	0	0	0	0	0	0	0	0	683
RL007_2	5	52,397	145	116	216	303	687	25	29	18	12	9	0	4	5,302	24	49	26	9	1	0	0	0	0	0	0	0	0	0	0	84
RL009_1	3	52,239	202	83	146	198	537	16	21	4	17	2	0	1	7,149	28	46	28	10	9	0	0	0	0	0	0	0	0	0	0	549
RL009_2	3	52,342	203	84	157	386	665	23	49	14	8	3	0	1	7,154	28	46	28	6	8	0	0	0	0	0	0	0	0	0	0	80
RL010_1	1	39,492	133	86	169	228	528	20	14	7	20	4	1	0	7,042	18	33	35	15	14	0	0	0	0	0	0	0	0	0	0	488
RL010_2	1	39,527	132	84	190	397	679	24	44	15	15	5	0	0	7,066	18	33	36	11	8	0	0	0	0	0	0	0	0	0	1	62
RL011_1	0	16,015	67	37	70	50	144	2	7	4	6	2	0	5	11,982	39	69	61	15	5	0	0	0	0	0	0	0	0	0	0	263
RL011_2	0	16,145	66	38	67	104	160	3	14	11	2	2	0	5	12,010	39	69	62	10	6	0	0	0	0	0	0	0	0	0	0	30

Supplementary Table S3. The number of sequenced reads of the voided urine specimens per sample

Sample	Number of reads				
	Input reads	Filtered reads	Denoised reads	Merged reads	Non-chimeric reads
DS065	56,457	16,011	15,928	14,810	5,937
DS067	31,014	8,509	8,494	8,417	2,254
DS078	381,338	305,706	305,467	287,743	80,457
DS500	68,263	48,776	48,714	41,401	17,694
DS623	50,002	3,353	3,324	2,949	2,058
KB001	46,551	1,044	1,018	399	399
KB008	37,588	4,170	4,109	3,830	2,101
KB011	45,056	3,399	3,372	2,996	1,486
KB012	68,706	1,491	1,454	925	797
RL004	42,919	3,360	3,346	2,771	1,864
RL007	59,274	5,974	5,956	5,763	3,648
RL009	61,210	7,741	7,723	6,926	4,399
RL010	48,284	7,661	7,577	7,095	4,708
RL011	28,820	12,154	12,027	11,138	6,853

Supplementary Table S4. Detected ASVs in urogenital tract and their taxonomic information

ASV_ID	Taxonomic rank	DS065	DS067	DS078	DS500	DS623	KB001	KB008	KB011	KB012	RL004	RL007	RL009	RL010	RL011
ASV01	d__Archaea; p__Halobacterota; c__Halobacteria; o__Halobacterales; f__Haloferacaceae; g__Haloplanus; s__Haloplanus_aerogenes	0	0	119	0	0	0	0	0	0	0	0	0	0	0
ASV02	d__Archaea; p__Halobacterota; c__Halobacteria; o__Halobacterales; f__Haloferacaceae; g__Haloplanus; s__Haloplanus_aerogenes	0	0	56	0	0	0	0	0	0	0	0	0	0	0
ASV03	d__Archaea; p__Halobacterota; c__Halobacteria; o__Halobacterales; f__Haloferacaceae; g__Haloplanus; s__Haloplanus_aerogenes	0	0	196	0	0	0	0	0	0	0	0	0	0	0
ASV04	d__Archaea; p__Halobacterota; c__Halobacteria; o__Halobacterales; f__Haloferacaceae; g__Haloplanus	0	0	176	0	0	0	0	0	0	0	0	0	0	0
ASV05	d__Archaea; p__Euryarchaeota; c__Methanobacteria; o__Methanobacteriales; f__Methanobacteriaceae; g__Methanobrevibacter	0	0	0	391	0	0	0	0	0	0	0	0	0	0
ASV06	d__Archaea; p__Halobacterota; c__Halobacteria; o__Halobacterales; f__Haloferacaceae; g__Haloplanus; s__Haloplanus_ruber	0	89	0	0	0	0	0	0	0	0	0	0	0	0
ASV07	d__Archaea; p__Halobacterota; c__Halobacteria; o__Halobacterales; f__Haloferacaceae; g__Haloplanus; s__Haloplanus_aerogenes	0	0	112	0	0	0	0	0	0	0	0	0	0	0
ASV08	d__Archaea; p__Crenarchaeota; c__Bathyarchaeia; o__Bathyarchaeia; f__Bathyarchaeia; g__Bathyarchaeia; s__archaeon_RBG_16_50_20	0	0	0	0	0	0	316	0	0	0	0	0	0	0
ASV09	d__Archaea; p__Crenarchaeota; c__Bathyarchaeia; o__Bathyarchaeia; f__Bathyarchaeia; g__Bathyarchaeia; s__archaeon_RBG_16_50_20	0	0	0	0	0	0	309	0	0	0	0	0	0	0
ASV10	d__Archaea; p__Crenarchaeota; c__Nitrososphaeria; o__Nitrososphaerales; f__Nitrososphaeraceae; g__Nitrososphaeraceae	0	0	0	0	0	0	0	0	0	0	461	0	0	0
ASV11	d__Archaea; p__Crenarchaeota; c__Nitrososphaeria; o__Nitrososphaerales; f__Nitrososphaeraceae; g__Candidatus_Nitrososphaera	0	0	0	0	0	0	0	0	0	148	0	0	0	0
ASV12	d__Archaea; p__Crenarchaeota; c__Nitrososphaeria; o__Nitrososphaerales; f__Nitrososphaeraceae; g__Nitrososphaeraceae	0	0	0	0	0	0	0	0	0	0	1,546	0	0	0
ASV13	d__Archaea; p__Halobacterota; c__Halobacteria; o__Halobacterales; f__Haloferacaceae; g__Haloplanus; s__Haloplanus_aerogenes	0	0	102	0	0	0	0	0	0	0	0	32	0	0
ASV14	d__Archaea	0	0	0	44	0	0	0	0	0	0	0	0	0	0
ASV15	d__Archaea; p__Crenarchaeota; c__Nitrososphaeria; o__Nitrosotaleales; f__Nitrosotaleaceae; g__Nitrosotaleaceae; s__uncultured_archaeon	0	0	0	0	0	0	0	155	0	0	0	0	0	0
ASV16	d__Archaea; p__Crenarchaeota; c__Nitrososphaeria; o__Nitrosopumilales; f__Nitrosopumilaceae; g__Candidatus_Nitrosotenuis; s__thaumarchaeote_enrichment	0	0	0	0	0	0	0	188	0	0	0	0	0	0
ASV17	d__Archaea; p__Halobacterota; c__Halobacteria; o__Halobacterales; f__Haloferacaceae; g__Halolamina	0	0	0	0	0	0	0	0	0	0	0	0	0	71
ASV18	d__Archaea; p__Halobacterota; c__Halobacteria; o__Halobacterales; f__Haloferacaceae; g__Haloplanus; s__Haloplanus_aerogenes	245	137	4,905	0	60	0	0	0	0	0	0	180	195	0
ASV19	d__Archaea; p__Halobacterota; c__Halobacteria; o__Halobacterales; f__Haloferacaceae; g__Haloplanus	0	0	177	0	0	0	0	0	0	0	0	0	0	0
ASV20	d__Archaea; p__Crenarchaeota; c__Nitrososphaeria; o__Nitrosopumilales; f__Nitrosopumilaceae; g__Candidatus_Nitrosotenuis; s__thaumarchaeote_enrichment	0	0	0	0	0	0	0	533	0	0	0	0	0	0
ASV21	d__Archaea; p__Halobacterota; c__Halobacteria; o__Halobacterales; f__Haloferacaceae; g__Haloplanus	0	0	224	0	0	0	0	0	0	0	0	0	0	0

ASV22	d__Archaea; p__Halobacterota; c__Halobacteria; o__Halobacterales; f__Haloferacaceae; g__Haloplanus; s__Haloplanus_aerogenes	0	0	185	0	0	0	0	0	0	0	0	0	0	0
ASV23	d__Archaea; p__Halobacterota; c__Halobacteria; o__Halobacterales; f__Haloferacaceae; g__Haloplanus; s__Haloplanus_aerogenes	0	0	444	0	0	0	0	0	0	0	0	75	58	0
ASV24	d__Archaea; p__Halobacterota; c__Halobacteria; o__Halobacterales; f__Haloferacaceae; g__Haloplanus	0	0	190	0	0	0	0	0	0	0	0	0	0	0
ASV25	d__Archaea; p__Crenarchaeota; c__Nitrososphaeria; o__Nitrososphaerales; f__Nitrososphaeraceae; g__Nitrososphaeraceae	0	0	0	0	0	0	0	0	0	0	495	0	0	0
ASV26	d__Archaea; p__Euryarchaeota; c__Methanobacteria; o__Methanobacteriales; f__Methanobacteriaceae; g__Methanosphaera; s__Methanosphaera_stadtmanae	0	0	0	3,511	0	0	0	0	0	0	0	0	0	0
ASV27	d__Archaea; p__Halobacterota; c__Halobacteria; o__Halobacterales; f__Haloferacaceae; g__Haloplanus	0	0	278	0	0	0	0	0	0	0	0	0	0	0
ASV28	d__Archaea; p__Halobacterota; c__Halobacteria; o__Halobacterales; f__Haloferacaceae; g__Haloplanus; s__Haloplanus_aerogenes	0	0	34	0	0	0	0	0	0	0	0	0	0	0
ASV29	d__Archaea; p__Halobacterota; c__Halobacteria; o__Halobacterales; f__Haloferacaceae; g__Haloplanus; s__Haloplanus_aerogenes	788	96	3,095	0	114	0	0	0	0	0	0	322	309	0
ASV30	d__Archaea; p__Halobacterota; c__Halobacteria; o__Halobacterales; f__Haloferacaceae; g__Haloplanus	0	0	527	0	0	0	0	0	0	0	0	0	0	0
ASV31	d__Archaea; p__Halobacterota; c__Halobacteria; o__Halobacterales; f__Haloferacaceae; g__Haloplanus; s__Haloplanus_aerogenes	510	0	0	0	0	0	0	0	0	0	0	0	0	0
ASV32	d__Archaea; p__Halobacterota; c__Halobacteria; o__Halobacterales; f__Haloferacaceae; g__Haloplanus	0	0	116	0	0	0	0	0	0	0	0	0	0	0
ASV33	d__Archaea; p__Halobacterota; c__Halobacteria; o__Halobacterales; f__Haloferacaceae; g__Haloplanus; s__Haloplanus_aerogenes	0	0	63	0	0	0	0	0	0	0	0	0	0	0
ASV34	d__Archaea; p__Halobacterota; c__Halobacteria; o__Halobacterales; f__Haloferacaceae; g__Haloplanus; s__Haloplanus_natans	0	0	136	0	0	0	0	0	0	0	0	0	0	0
ASV35	d__Archaea; p__Halobacterota; c__Halobacteria; o__Halobacterales; f__Haloferacaceae; g__Haloplanus	0	0	426	0	0	0	0	0	0	0	0	0	0	0
ASV36	d__Archaea; p__Halobacterota; c__Halobacteria; o__Halobacterales; f__Haloferacaceae; g__Haloplanus; s__Haloplanus_aerogenes	0	0	51	0	0	0	0	0	0	0	0	0	0	0
ASV37	d__Archaea; p__Euryarchaeota; c__Methanobacteria; o__Methanobacteriales; f__Methanobacteriaceae; g__Methanobrevibacter	0	0	0	2,341	0	0	0	0	0	0	0	0	0	0
ASV38	d__Archaea; p__Halobacterota; c__Halobacteria; o__Halobacterales; f__Haloferacaceae; g__Haloplanus; s__Haloplanus_aerogenes	894	351	11,343	0	229	0	0	0	0	0	0	567	568	0
ASV39	d__Archaea; p__Crenarchaeota; c__Nitrososphaeria; o__Nitrosopumilales; f__Nitrosopumilaceae; g__Candidatus_Nitrosotenuis; s__thaumarchaeote_enrichment	0	0	0	0	0	0	0	247	0	0	0	0	0	0
ASV40	d__Archaea; p__Halobacterota; c__Halobacteria; o__Halobacterales; f__Haloferacaceae; g__Haloplanus; s__Haloplanus_aerogenes	102	0	0	0	0	0	0	0	0	0	0	0	0	0
ASV41	d__Archaea; p__Euryarchaeota; c__Methanobacteria; o__Methanobacteriales; f__Methanobacteriaceae; g__Methanobrevibacter	0	0	0	109	0	0	0	0	0	0	0	0	0	0
ASV42	d__Archaea; p__Halobacterota; c__Halobacteria; o__Halobacterales; f__Haloferacaceae; g__Haloplanus; s__Haloplanus_aerogenes	0	0	34	0	0	0	0	0	0	0	0	0	0	0
ASV43	d__Archaea; p__Halobacterota; c__Halobacteria; o__Halobacterales; f__Haloferacaceae; g__Haloplanus; s__Haloplanus_aerogenes	1,646	714	19,247	0	719	0	0	0	0	0	0	1,606	1,747	0
ASV44	d__Archaea; p__Crenarchaeota; c__Bathyarchaeia; o__Bathyarchaeia; f__Bathyarchaeia; g__Bathyarchaeia; s__archaeon_RBG_16_50_20	0	0	0	0	0	0	806	0	0	0	0	0	0	0

ASV45	d__Archaea; p__Halobacterota; c__Halobacteria; o__Halobacterales; f__Haloferacaceae; g__Haloplanus	0	0	267	0	0	0	0	0	0	0	0	0	0	0
ASV46	d__Archaea; p__Crenarchaeota; c__Nitrososphaeria; o__Nitrosopumilales; f__Nitrosopumilaceae; g__Candidatus_Nitrosotenuis; s__thaumarchaeote_enrichment	0	0	0	0	0	0	0	127	0	0	0	0	0	0
ASV47	d__Archaea; p__Halobacterota; c__Halobacteria; o__Halobacterales; f__Haloferacaceae; g__Haloplanus	0	0	31	0	0	0	0	0	0	0	0	0	0	0
ASV48	d__Archaea; p__Halobacterota; c__Halobacteria; o__Halobacterales; f__Haloferacaceae; g__Haloplanus; s__Haloplanus_aerogenes	450	507	26,293	0	226	0	0	0	0	0	0	656	595	0
ASV49	d__Archaea; p__Halobacterota; c__Halobacteria; o__Halobacterales; f__Haloferacaceae; g__Halolamina	0	0	0	0	0	0	0	12	58	81	0	0	0	1,164
ASV50	d__Archaea; p__Halobacterota; c__Halobacteria; o__Halobacterales; f__Haloferacaceae; g__Haloplanus; s__Haloplanus_aerogenes	1,302	344	10,252	0	342	0	0	0	0	0	0	820	828	0
ASV51	d__Archaea; p__Halobacterota; c__Halobacteria; o__Halobacterales; f__Haloferacaceae; g__Halolamina	0	0	0	0	0	250	0	0	271	0	0	0	0	2,332
ASV52	d__Archaea; p__Crenarchaeota; c__Nitrososphaeria; o__Nitrososphaerales; f__Nitrososphaeraceae; g__Candidatus_Nitrososphaera	0	0	0	0	0	0	0	0	0	400	0	0	0	0
ASV53	d__Archaea; p__Euryarchaeota; c__Methanobacteria; o__Methanobacteriales; f__Methanobacteriaceae; g__Methanobrevibacter	0	0	0	4,169	0	0	0	0	0	0	0	0	0	0
ASV54	d__Archaea; p__Euryarchaeota; c__Methanobacteria; o__Methanobacteriales; f__Methanobacteriaceae; g__Methanobrevibacter	0	0	0	1,041	0	0	0	0	0	0	0	0	0	0
ASV55	d__Archaea; p__Halobacterota; c__Halobacteria; o__Halobacterales; f__Haloferacaceae; g__Halolamina	0	0	0	0	0	0	0	0	0	0	0	0	0	298
ASV56	d__Archaea; p__Crenarchaeota; c__Bathyarchaeia; o__Bathyarchaeia; f__Bathyarchaeia; g__Bathyarchaeia; s__archaeon_RBG_16_50_20	0	0	0	0	0	0	425	0	0	0	0	0	0	0
ASV57	d__Archaea; p__Crenarchaeota; c__Nitrososphaeria; o__Nitrosopumilales; f__Nitrosopumilaceae; g__Candidatus_Nitrosotenuis; s__thaumarchaeote_enrichment	0	0	0	0	0	0	0	55	0	0	0	0	0	0
ASV58	d__Archaea; p__Halobacterota; c__Halobacteria; o__Halobacterales; f__Haloferacaceae; g__Haloplanus	0	0	201	0	0	0	0	0	0	0	0	0	0	0
ASV59	d__Archaea; p__Euryarchaeota; c__Methanobacteria; o__Methanobacteriales; f__Methanobacteriaceae; g__Methanobrevibacter	0	0	0	959	0	0	0	0	0	0	0	0	0	0
ASV60	d__Archaea; p__Euryarchaeota; c__Methanobacteria; o__Methanobacteriales; f__Methanobacteriaceae; g__Methanobrevibacter	0	0	0	394	0	0	0	0	0	0	0	0	0	0
ASV61	d__Archaea; p__Crenarchaeota; c__Nitrososphaeria; o__Nitrosopumilales; f__Nitrosopumilaceae; g__Candidatus_Nitrosotenuis; s__thaumarchaeote_enrichment	0	0	0	0	0	0	0	169	0	0	0	0	0	0
ASV62	d__Archaea; p__Halobacterota; c__Halobacteria; o__Halobacterales; f__Haloferacaceae; g__Halolamina	0	0	0	0	0	0	0	0	0	0	0	0	0	395
ASV63	d__Archaea; p__Halobacterota; c__Halobacteria; o__Halobacterales; f__Haloferacaceae; g__Haloplanus; s__Haloplanus_aerogenes	0	0	228	0	0	0	0	0	0	0	0	0	0	0
ASV64	d__Archaea; p__Halobacterota; c__Halobacteria; o__Halobacterales; f__Haloferacaceae; g__Haloparvum; s__uncultured_archaeon	0	0	0	0	0	0	0	0	0	0	0	0	18	0
ASV65	d__Archaea; p__Crenarchaeota; c__Nitrososphaeria; o__Nitrososphaerales; f__Nitrososphaeraceae; g__Candidatus_Nitrososphaera	0	0	0	0	0	0	0	0	0	232	0	0	0	0
ASV66	d__Archaea; p__Euryarchaeota; c__Methanobacteria; o__Methanobacteriales; f__Methanobacteriaceae; g__Methanobrevibacter	0	0	0	4,510	0	0	0	0	0	0	0	0	0	0
ASV67	d__Archaea; p__Halobacterota; c__Halobacteria; o__Halobacterales; f__Haloferacaceae; g__Halolamina	0	0	0	0	0	0	0	0	0	0	0	0	0	1,142

ASV68	d__Archaea; p__Crenarchaeota; c__Nitrososphaeria; o__Nitrososphaerales; f__Nitrososphaeraceae; g__Candidatus_Nitrososphaera	0	0	0	0	0	0	0	0	0	686	0	0	0	0
ASV69	d__Archaea; p__Halobacterota; c__Halobacteria; o__Halobacterales; f__Haloferacaceae; g__Haloplanus	0	0	412	0	0	0	0	0	0	0	0	0	0	0
ASV70	d__Archaea; p__Halobacterota; c__Halobacteria; o__Halobacterales; f__Haloferacaceae; g__Haloplanus; s__Haloplanus_aerogenes	0	0	227	0	0	0	0	0	0	0	0	0	0	0
ASV71	d__Archaea; p__Halobacterota; c__Halobacteria; o__Halobacterales; f__Haloferacaceae; g__Haloplanus; s__Haloplanus_aerogenes	0	0	61	0	0	0	0	0	0	0	0	0	0	0
ASV72	d__Archaea; p__Halobacterota; c__Halobacteria; o__Halobacterales; f__Haloferacaceae; g__Halolamina	0	0	0	0	0	0	0	0	0	0	0	0	0	1,437
ASV73	d__Archaea; p__Halobacterota; c__Halobacteria; o__Halobacterales; f__Haloferacaceae; g__Haloplanus	0	0	197	0	0	0	0	0	0	0	0	0	0	0
ASV74	d__Archaea; p__Crenarchaeota; c__Nitrososphaeria; o__Nitrososphaerales; f__Nitrososphaeraceae; g__Nitrososphaeraceae	0	0	0	0	0	0	0	0	0	0	669	0	0	0
ASV75	d__Archaea; p__Crenarchaeota; c__Nitrososphaeria; o__Nitrososphaerales; f__Nitrososphaeraceae; g__Candidatus_Nitrososphaera	0	0	0	0	0	0	0	0	0	236	0	0	0	0

Supplementary Table S5. The sequence similarity between the urinary archaeal ASVs and previously isolated type or candidatus strains in the same genus

**Genus**	**Sequence similarity (%)**
Halolamina	97·9–99·3 (98·7±0·5)
Haloplanus	94·4–99·3 (97·3±1·1)
Methanobrevibacter	97·2–99·3 (98·4±0·7)
Unclassified Bathyarchaeia	98·6–99.3 (99.0±0.4)
Unclassified Nitrososphaeraceae	98.6–99.3 (98.9±0.4)
'*Candidatus* Nitrososphaera'	98.6–99.3 (98.9±0.3)
'Candidatus Nitrosotenuis'	97.9–99.3 (98.7±0.6)
